# Hybrid and Secure E-Health Data Sharing Architecture in Multi-Clouds Environment

**DOI:** 10.1007/978-3-030-51517-1_21

**Published:** 2020-05-31

**Authors:** Tayssir Ismail, Haifa Touati, Nasreddine Hajlaoui, Hassen Hamdi

**Affiliations:** 8 Digital Research Centre of Sfax, Sfax, Tunisia; 9 Institut Mines-Télécom, CNRS, Paris, France; 10 Université de Sherbrooke, Sherbrooke, QC Canada; 11 Digital Research Centre of Sfax, Sfax, Tunisia; 12 University of Sfax, Sfax, Tunisia; 13 Hatem Bettahar Research Unit IResCoMath, Gabes, Tunisia; 14 Faculty of Science of Gabes, Gabes, Tunisia; 15 MIRACL Laboratory, FSEG-Sfax, Sfax, Tunisia

**Keywords:** E-health system security, Privacy preservation, Multi-cloud, Data storage, Data share, Data encryption

## Abstract

Healthcare is among the sectors showing efforts in adopting cloud computing to its services considering the provided cost reduction and healthcare process efficiency. However, outsourcing patient’s sensitive data increases the concerns regarding security, privacy, and integrity of healthcare data. Therefore, there is a need for building a trust relationship between patients and e-health systems. In this paper, we propose a privacy-preserving framework, called Hybrid and Secure Data Sharing Architecture (HSDSA), to secure data storage in e-health systems. Our approach improves security in healthcare by maintaining the privacy and confidentiality of sensitive data and preventing threats. In fact, in the upload phase, Multi-cloud environment is used to store Rivest–Shamir–Adleman (RSA) encrypted medical records. We adopt a Shamir’s secret sharing approach for the distribution of shares to different independent cloud providers. In the retrieval phase, the reconstruction operation is based on the (*t*, *n*) strategy. To check the requester identity and to prove the hash possession, we used a zero-knowledge cryptography algorithm, namely the Schnorr algorithm. The patient has a total control over the generation and management of the decryption keys using Diffie-Hellman algorithm without relying on a trusted authority.

## Introduction

Cloud computing is a new promising technology that leverages the user from the burden of hardware maintenance and offers dynamically flexible and scalable computational resources accessible from any place where a network is available. The emergence of this paradigm has deeply influenced many domains and especially the healthcare sector. However, the usage of this model in the healthcare domain needs the reinforcement of security measures because data are susceptible to lose, leakage or theft. Therefore, confidentiality and integrity of the stored Electronic Health Records (EHR) are deemed as one of the major challenges elevated by the external storage. Besides, the privacy of sensitive data must be guaranteed. To overcome the above cited challenges, cryptographic techniques for securing e-health systems are widely adopted. But the reliance on a single cloud storage provider has shown many drawbacks like a single point of failure, vendor lock-in and malicious insiders. To narrow down the listed disadvantages, it is advisable to use multi-cloud architecture. One of the key concepts of this model is to store data on different cloud server providers where an insider is not able to reconstruct the original data from a single share [1].

In this context, several solutions have been proposed in the literature to ensure secure multi-cloud storage in e-health systems [2–5]. They mainly have two phases: *storage* and *retrieval*. They also all use cryptographic primitives to ensure EHRs security. Authors of [2] use an Attribute Based-Encryption (ABE) for selective access authorisation and cryptographic secret sharing. The EHRs split and reconstruction is done through a proxy. In [3], ABE is used for selective data sharing with physicians without allowing them to know the precise description of the patient’s illnesses. Biometrics based authentication and Kerberos tickets session are used in [4] to guarantee secure interaction with the EHR system. In addition, a steganographic technique is used to store EHR. In [5], authors propose the use of Shamir’s Secret Sharing not only to distribute EHR shares among cloud servers but to retrieve the requested EHR from partial cloud servers. In summary, the main drawback of [2–5] is the reliance on a trusted third party which may not be adequate for practical use as they show security risks. Hence, a secure privacy-preserving data storage solution is still needed to improve the patient role to monitor his data on the cloud.

In this paper, we present a Hybrid and Secure Data Sharing Architecture (HSDSA), for secure and privacy-preserving storing and sharing of patient’s sensitive data in a Multi-cloud environment without relying on a trusted third party. In HSDSA, cloud providers are assumed to be semi-trusted: honest but curious. HSDSA gives the patient total control over the generation and management of the decryption keys without relying on a trusted authority and thus it is more applicable for public cloud environments. To protect the data from external attackers, Rivest–Shamir–Adleman (RSA) encryption is applied before outsourcing EHR. To secure data against cloud providers curiosity, Shamir’s secret sharing is adopted. The resulted shares are distributed to multiple clouds. To download an EHR, HSDSA recovers its shares using an outsourcing reconstruction operation based on the (*t*, *n*) strategy. To complete the file decryption, a Schnorr-based technique is used to prove data possession and to verify the requester identity. Then a session, using the Diffie Hellman (DH) algorithm, is created to securely exchange the decryption key. Finally, the key is extracted and the original EHR could be recovered. Outsourcing reconstruction operation based on the (*t*, *n*) strategy is used.

The remainder of the paper is organised as follows. Section 2 gives an overview of the overall architecture of the proposed framework and its components. Sections 3 and 4 detail the different techniques used in the storage and retrieval processes. Finally, Sect. 5 concludes the paper and highlights some open issues.

## Architecture Overview of the Proposed Scheme

**Fig. 1.**
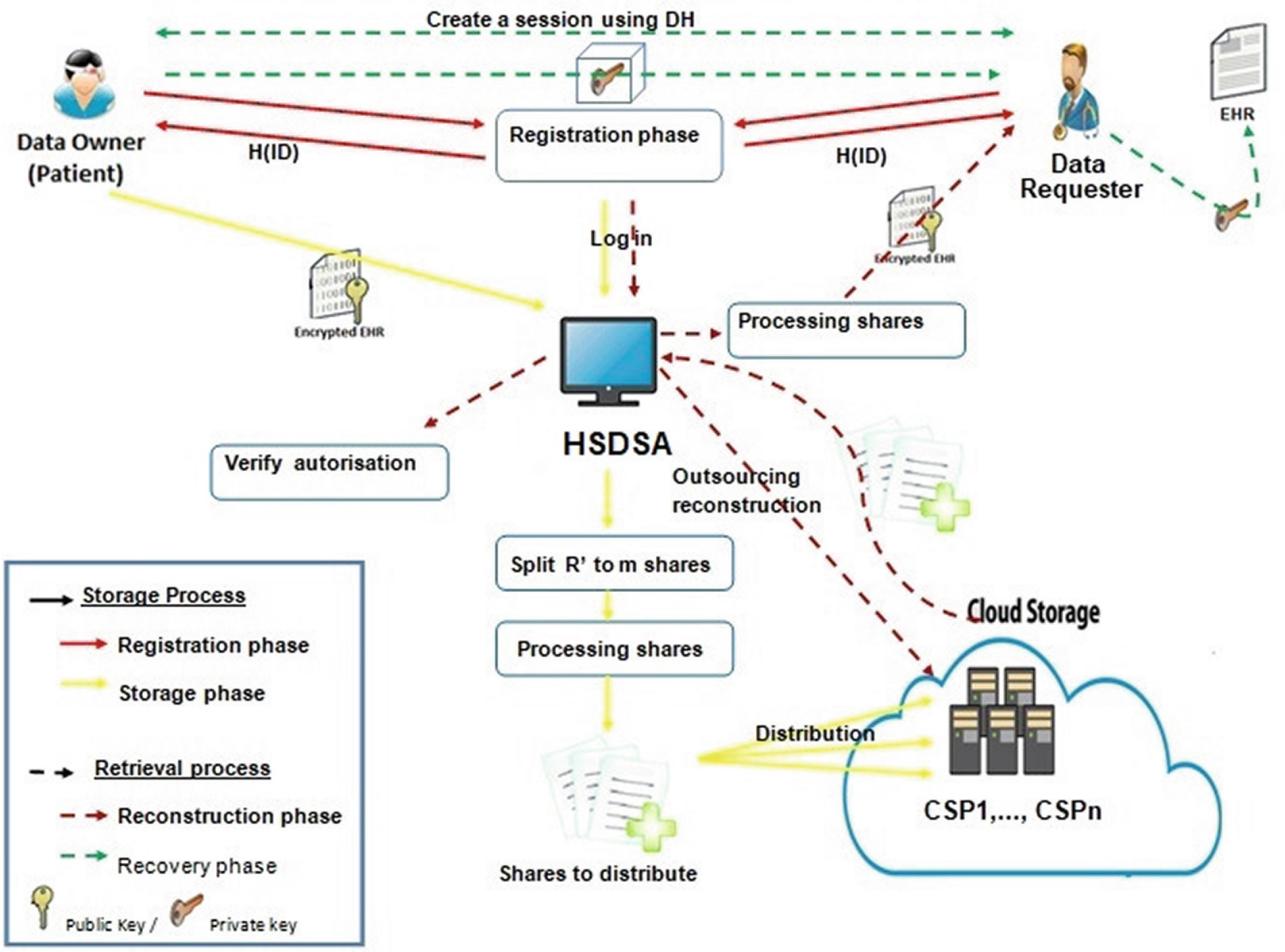
Architecture overview

We recall that our goal is to securely store EHRs in multi-cloud environment and to securely share them among healthcare organisations staff. In the following, we will give an overview of the HSDSA framework in which we focus on the context of medical data storage, share and retrieval. The basics of HSDSA are shown in Fig. 1. Our system compromises three different entities: Data Owner (DO), Data Requester (DR) and *n* CSP ($CSP_{1}$,...,$CSP_{n}$). The key components of the HSDSA framework include:

- **The storage process** which is composed of two phases, namely the **registration** phase and the **storage** phase.
- **The retrieval process** which is composed of two phases, namely the EHR **reconstruction** and the EHR **recovery** phases.

As shown in Fig. 1, the workflow of HSDSA is as follows:

- **The registration phase** starts when a DO or a DR signs in to the Framework Interface. After being signed to HSDSA, a DO or a DR receives in response the hash of his identity H($ID_{DO,DR}$).
- **The storage phase** starts when a DO wants to store his EHRs, he calculates the digital signature of his EHR (*R*), encrypts *R* using Rivest–Shamir–Adleman (RSA) algorithm for both and logs in to the HSDSA. Then, the DO uploads his encrypted record ($R^{'}$) and the hash value of the original record H(R). HSDSA generates a unique identifier ($ID_{R}$) of the EHR to guarantee the anonymity of stored data in Cloud servers and stores H($ID_{DO}$), H(R) and $ID_{R}$. The framework calculates the hash of the uploaded EHR ($R^{'}$) and splits it into *m* shares. Then, it performs an exclusive OR operation between each share ($S^{'}_{i}$) and the hash value of $R^{'}$. The distribution is done using Shamir’s secret sharing algorithm and the resulted shares are sent to *n* different Cloud Server Providers $CSP_{1}$, ... $CSP_{n}$.
- **The reconstruction phase** starts when a DO or an authorised user DR wants to get the EHR, he sends a request to the framework. After confirming the request, HSDSA assigns a CSP ($CSP_{R}$) to perform the reconstruction step. The $CSP_{R}$ gets *t* shares or more from $CSP_{1}$, ... $CSP_{n}$. Once the reconstruction is done, the $CSP_{R}$ returns the resulted shares to the framework.
- Finally, **the recovery phase**, when the DR wants to get the DO’s private key, he has to prove that he is the right DR and he has the correct hash value of *R*. To this end, the Schnorr algorithm is used. Once, the DO makes sure that the DR is an authorised requester and that he possesses the encrypted version of the EHR ($$R^{'})$$, then the DR and the DO try to establish a session using Diffie-Hellman (DH) algorithm to exchange decryption key securely. Once they agree on a session key, $$ K_{s} $$. The DO encrypts his private key using $$K_{s}$$ and sends it to the DR. Once the private key is extracted, the DR can finally recover the desired EHR (R).

## Analysis of the Proposed Storage Process

In the following, we detail the techniques used in the two phases of the storage process: the registration phase and the storage phase.

### The Registration Phase

As recommended in cloud-based storage solutions, building a trust relationship between partners is a necessity. To achieve this goal, the first step is to make sure that all users are registered to the framework. If a new user wants to benefit from services provided by the HSDSA framework, he must be correctly authenticated. Once he registers, he receives a value containing the hash of his identity H($ID_{DO,DR}$) in order to maintain the anonymity of user identities.

### The Storage Phase

HSDSA acts as an intermediary between DO and CSPs. Our goal is to provide a secure storage facility to authorised users. This phase involves Shamir’s secret sharing technique to make sure that the multi-cloud environment, used to store shares, is a collusion-safe. By collusion-safe we mean that if two or more CSPs combine their keys, they cannot decrypt the data. Ten steps, illustrated in Table 1, describe the storage phase.

**Table 1.**
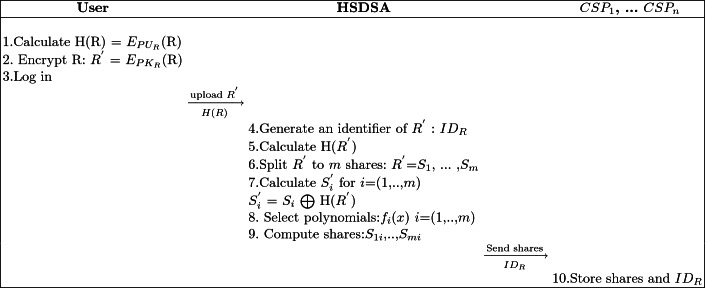
Scenario of the storage phase

When a DO wants to store an EHR, he calculates the digital signature of the original EHR (R). Then the RSA is used to split the selected EHR into blocks and encrypt them. Sequential execution of RSA needs a lot of calculation. Therefore, we use the enhancement proposed in [6] where authors have parallelized the process of encryption and decryption of a large number of data blocks. The resulted file $R^{'}$ and H(R) are sent to HSDSA.

1 $$\begin{aligned} R^{'} = E_{PK_{R}}(R) \end{aligned}$$

When the framework receives $R^{'}$ and H(R), it generates a unique identifier $ID_{R}$ corresponding to the file $R^{'}$. This is used to guarantee the unlinkability between DO and EHR. After that, HSDSA computes the hash of $R^{'}$ (H($R^{'}$)) and stores $ID_{R}$, H(R) and H($R^{'}$). Next the framework splits $R^{'}$ into *m* shares [$S_{1}$, ... ,$S_{m}$], performs the exclusive OR operation of each split of $R^{'}$ with H($R^{'}$).

2 $$\begin{aligned} \begin{array}{r c l} [S^{'}_{1},...,S^{'}_{m}] &{} = &{} R^{'} \bigoplus H(R^{'})\\ &{} = &{} [S_{1}, ... ,S_{m}] \bigoplus H(R^{'})\\ &{} = &{} [S_{1}] \bigoplus H(R^{'}), .., [S_{m}] \bigoplus H(R^{'})\\ \end{array} \end{aligned}$$

[$S^{'}_{1}$, ... ,$S^{'}_{m}$] are the shares to be stored in independent CSPs. To securely distribute the shares, we adopted Shamir’s secret sharing protocol. It represents a so-called (*t*,*n*) threshold scheme with 1 $\le$* t* $\le$ *n*. This mechanism permits the distribution of a document among *n* parts in a way that reconstruction is possible if at least *t* shares are present. Suppose a share $S^{'}_{i}$ (for *i* i = 1 ... *m*), Shamir’s secret sharing algorithm sets $a_{i0}$ = $S^{'}_{i}$, chooses $a_{i1}$, ..., $a_{it-1}$ at random, takes distinct values $x_{1}$, $x_{2}$,..., $x_{m}$ with *m* $\ge$ *t*-1 and computes the shares to distribute, as follows:

$$\left\{ \begin{array}{c} S_{1i} = (x_{i},f_{1}(x_{i})) \\ ....\\ S_{mi} = (x_{i},f_{m}(x_{i}))\\ \end{array} \right. \ , \ for \ i=1..n$$

In the proposed architecture, HSDSA selects *m* polynomials.

$$\left\{ \begin{array}{c} f_{1}(x)= a_{10}+ a_{11}x+ a_{12}x^{2}+ ...+ a_{1t-1}x^{t-1} \ mod\ p\\ ...\\ f_{m}(x)= a_{m0}+ a_{m1}x+ a_{m2}x^{2}+...+ a_{mt-1}x^{t-1}\ mod\ p\\ \end{array} \right. $$

Where

$$\begin{aligned} \begin{array}{r l} \begin{bmatrix} a_{11},...,a_{1t-1}\\ ...\\ a_{m1}, ..., a_{mt-1}\\ \end{bmatrix} &{} \in \quad \mathbb {Z_p}\\ \end{array} \end{aligned}$$

The HSDSA computes *n* shares $S_{1i}$, ..., $S_{mi}$ (i = 1, ..., n) and distributes them to $CSP_{1}$, ..., $CSP_{n}$.

## Analysis of the Proposed Retrieval Process

File retrieval, also known as file reconstruction, is the reversal process of file distribution and file slicing. In this framework the retrieval process starts when a data requester DR needs to get an EHR. He must log in and submit the EHR identifier ($ID_{R}$). HSDSA checks if the DR has the right to get the requested EHR. If the authorisation succeeded, then the reconstruction phase starts.

### The Reconstruction Phase

Since the reconstruction of $R^{'}$ requires a massive amount of computation and that client resources are limited, we will use the reconstruction outsourcing scheme proposed in [5]. The framework assigns a CSP ($CSP_{R}$) to reconstruct shares $S^{'}_{j}$. The reconstruction is considered successfull only if $CSP_{R}$ gets at least *t* shares from $CSP_{1}$, ..., $CSP_{n}$. We assume that $CSP_{R}$ gets *k* shares.

$$\begin{aligned} \begin{bmatrix} S_{11},...S_{m1}\\ ...\\ S_{1k},...,S_{mk}\\ \end{bmatrix}, (k \ge t) \end{aligned}$$

$CSP_{R}$ computes $S^{'}_{j}$ for $j=(1,...,m)$ using Lagrange interpolation polynomial and sends them to HSDSA:

3 $$\begin{aligned} S^{'}_{j} = \sum _{i=1}^{k} S_{ji}\prod _{l=1,l\ne i}^k \frac{x_{i}}{x_{i}-x_{l}} \ mod \ p \ (j=1,...,m) \end{aligned}$$

To make sure that $CSP_{R}$ could not reveal any useful information, knowing that he is a curious and dishonest party, doing an exclusive OR operation helps to blind the content. Upon receipt of the shares, the HSDSA framework performs the exclusive OR operation between $S^{'}_{j}$ and H($R{'}$) to get $S_{j}$.

### The Recovery Phase

This phase aims to securely transfer the Data Owner’s private key to the right Data Requester. Table 2 illustrates the main steps related to this phase.

**Table 2.**
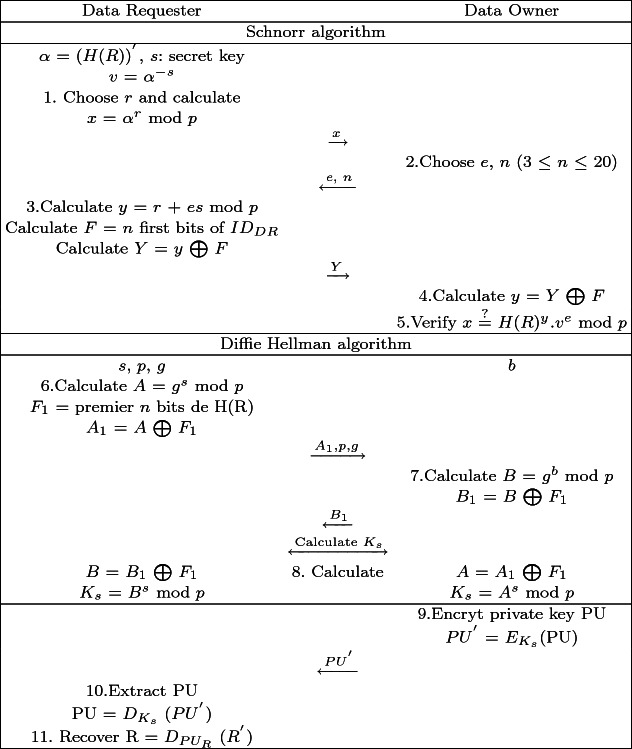
Scenario of the recovery phase

Once the reconstruction phase is done, HSDSA sends $ID_{DO}$/$ID_{DR}$ to the Data Owner and to the Data Receiver. First the DR has to prove to the DO that he holds the right hash value of the original file $(H(R))^{'}$ and that he is the correct DR. For this purpose, a Schnorr’s identification protocol [7] is used not only to prove the hash possession but also to verify the DR identity. In the process of the latter algorithm the DO checks if H(R) ${\mathop {=}\limits ^{?}}$ ($H(R))^{'}$), and verifies if the *n* first bits of the DR identity match the $ID_{DR}$ previously sent by FI. Then the DO and the DR must establish a secure connection for key exchange, based on Diffie-Hellman (DH) scheme Ephemeral version [8], reinforced with the hash value of the original file (H(R)). Establishing a session means that the two partners have agreed on session key ($K_{s}$) that will be used to crypt partners metadata. Next, the DO encrypts his private key (PU) using $K_{s}$ and sends the resulted value $PU^{'}$ to the DR.

4 $$\begin{aligned} PU^{'}= E_{K_{s}}(PU) \end{aligned}$$

The DR decrypts $PU^{'}$ using $K_{s}$ to extract the DO private key. Possessing PU, the DR decrypts $R^{'}$ to finally recover the original EHR (R).

5 $$\begin{aligned} PU = D_{K_{s}}(PU^{'}) \end{aligned}$$

6 $$\begin{aligned} R = D_{PU}(R^{'}) \end{aligned}$$

## Conclusion

In this paper, we presented HSDSA, a novel architecture for secure EHR. HSDSA includes several techniques, namely (i) RSA algorithm to guarantee the security of outsourced data, (ii) Shamir’s secret sharing to securely distribute data across multiple clouds, (iii) a secure outsourcing reconstruction based on the (*t*, *n*) strategy, (iv) a Schnorr-based technique to prove data possession and to verify the requester identity and (v) a Diffie-Hellman algorithm to securely exchange decryption key. The proposed scheme allows the patient to get total control over the generation and management of the decryption keys without relying on a trusted authority. In a future work, we plan to add governmental organisation as Data Requester. These latter need to access data without the Data Owner authorisation. Hence, we aim to protect privacy while giving them access to EHR.
